# Culinary medicine in medical education: a pilot study targeting cancer risk reduction strategies through culinary and lifestyle medicine education

**DOI:** 10.3389/fnut.2025.1549388

**Published:** 2025-04-25

**Authors:** Stephanie R. Harris, Elaine Borawski, Ryanne Lachman, Lindsay Malone, Jessica DePalma, Hope Barkoukis

**Affiliations:** Department of Nutrition, School of Medicine, Case Western Reserve University, Cleveland, OH, United States

**Keywords:** culinary medicine, lifestyle medicine, medical education, cancer risk reduction, cancer prevention, medical school, chronic disease risk reduction, health education

## Abstract

**Introduction:**

Culinary Medicine (CM) is an avenue for interdisciplinary nutrition education intervention utilizing the expertise of dietitians, physicians, and other health care professionals (HCP). Despite the positive impacts that CM interventions can have on health, physician CM knowledge is lacking due in part to inadequate nutrition education in medical school curriculum. CM as a nutrition education modality promotes health and disease management for patients and providers, so it is critical to increase competency in CM. This pilot study evaluated the impact of a cancer prevention specific CM curriculum on medical students’ (i) cancer risk reduction (CRR) knowledge, (ii) CRR assessment/counseling attitudes and self-efficacy in clinical care, and (iii) personal health behaviors and cooking skills.

**Methods:**

Thirty-one 2nd year medical students (CALM students) participated in seven, 3-hour CRR focused CM education sessions and were compared to 55 non-enrolled students (control group). Education sessions incorporated a lecture, learning activity, and cooking experience focused on topics including dietary patterns, gut health, inflammation, metabolic health, hormone balance, environmental exposures, and prevention in practice/at home. A 46-item online pre-test (09/23) and post-test (03/24) survey assessed standardized measures of general nutrition/cancer knowledge, attitudes/beliefs, perceived control and self-efficacy around CRR diet/ lifestyle modifications; and intentions of integrating CRR strategies in practice.

**Results:**

78 students (91%) completed both surveys and the findings indicate that CALM students showed significant improvement over their peers in knowledge scores (*β* = 0.265, *t* = 2.14, *p* < 0.05), attitudes toward nutrition in the clinical setting (*β* = 0.203, *t* = 2.00, *p* < 0.05) and confidence in integrating CRR strategies in patient care (*β* = 0.401, *t* = 4.05, *p* < 0.001). Most significant changes occurred in confidence of being able to make a CRR plan and follow through with patients on the plan (*p* < 0.001).

**Discussion:**

This pilot study is among the first to incorporate and evaluate CRR-specific CM competencies in medical education. Given that the lifetime risk for developing cancer is high for Americans (~40%), education and implementation of CRR strategies among patients and providers must be emphasized. If research continues to demonstrate curriculum success in future cohorts, it is an innovative approach to teaching nutrition and CM competencies to HCP that is applicable to numerous disease states.

## 1 Introduction

The burden of cancer in the United States is significant. Americans hold a 40% lifetime risk of developing cancer, and with an estimated two million new diagnoses in 2024, millions of Americans will watch a loved one suffer this year (1). Genetic predisposition and epigenetic influences contribute to this risk, ranking cancer among the top 10 most prevalent chronic diseases and the second most likely cause of death in the US (1). Additionally, those who have contracted a chronic disease are also at an increased risk for cancer. Research among patients with a chronic disease found a 20% increased risk for cancer incidence and 30% increased risk of cancer mortality over 9 years; moreover, the research notes that 77% of patients diagnosed with cancer have one or more chronic diseases (2). Furthermore, the cost of cancer is staggering, as the initial treatment phase alone costs an average of $41,800 (3). By 2030, the national cost of cancer is expected to be $246 billion – and that only accounts for the rising costs of healthcare, not due to the expected rise in incidence of 6 of the 10 most prevalent cancers: breast, uterine, pancreas, prostate, kidney, and melanoma (1, 3).

The American Institute for Cancer Research (AICR) and the World Cancer Research Fund emphasize personal responsibility in reducing cancer risks, citing that the risk of 30–40% of cancers can be minimized through lifestyle changes such as decreasing tobacco usage, increasing physical activity, maintaining a healthy weight, and eating a balanced, healthy diet (4–6). Targeting these behaviors is critical to reducing cancer risk overall, including the risk of recurrence among survivors (4–7). Due to the complexities of cancer, there’s no clear prevention strategy. Instead, we turn to risk reduction strategies to avoid provoking cancer initiation, minimize vulnerability due to poor health status, identify early stages, and act to deter cancer development and progression.

More studies are needed to further elucidate the links between dietary choices and cancer risk (5). However, current research is sufficient to positively associate obesity (high BMI), central adiposity, and nearly all characteristics of the standard American diet including alcohol, processed meats, high-calorie, high-fat and high glycemic index diets, and low fruit and vegetable intake with increasing cancer risk (4, 5, 8–13). When evaluating the impact of dietary interventions on cancer, research has shown improvements in breast cancer pCR (14); T cell regulation (15); perioperative body composition in [gastrointestinal] cancer patients (16); and decreased inflammation with support from healthful plant-based diets (17), containing omega 3 fatty acids (15, 18), fiber, and polyphenols (18). One such plant-based dietary pattern that includes healthy fats (omega-3 fatty acids), limited meat intake, and regular intake of fruits and vegetables is the Mediterranean dietary pattern, which is associated with a decrease in cancer mortality (4–7, 15, 18, 19).

While research demonstrates dietary modifications are associated with cancer risk reduction (1, 4, 6, 9, 19), the question remains; how do we encourage behavior change? Culinary medicine (CM) is a strategy for nutrition intervention that promotes collaboration between healthcare professionals such as physicians and registered dietitian nutritionists (RDNs) to treat and prevent chronic illness with dietary interventions (20, 21). The field seeks to inform strategies and skills for healthy eating, from planning to cooking, thereby placing the power to prevent illness in the patient’s hands (20, 21). Studies demonstrate improvements in diabetes management, hypertension, BMI, cholesterol, self-efficacy, attitudes toward cooking, mental health, and quality of life in patients who engage with CM interventions (20, 22–24). Additionally, patients learning to prepare healthful meals also showed greater adherence to a Mediterranean dietary pattern and improved overall fruit and vegetable intake (20, 22, 23). For cancer risk reduction, these skills and behaviors are especially important considering both the Mediterranean dietary pattern and adequate intake of fruits and vegetables, are protective against cancer development (4, 9, 14–20).

However, before these skills and knowledge can be passed to patients, educating the healthcare providers is essential (20, 21, 25). Currently, the state of nutrition education for providers is rather bleak, as only 29% of medical schools provide the recommended 25 h of nutrition education (26). As a result, most attending physicians and residents do not have the foundational education to provide basic nutritional and lifestyle counseling to patients who need it and may not have access to easily refer to a registered dietitian nutritionist (26). CM education interventions taught in teaching kitchens provide an avenue to mitigate this lack of knowledge and confidence (27), with a growing number of medical schools beginning to incorporate this training through electives, service-learning opportunities, specialty tracks, and interest groups (21, 28). Medical students and residents who receive CM education interventions have reported improvements in their ability and confidence to advise and their patients nutritionally (26, 29–31). Additionally, medical students have expressed an interest in adjusting their dietary patterns (29–31), and have improved fruit and vegetable intake (31, 32) after CM education. Thus, CM education interventions benefit not only the patients, but also the providers (26, 29, 32, 33).

To date, none of the CM education offerings provided to medical students specifically target cancer risk reduction (21, 26). Thus, future providers ultimately lack the knowledge necessary to inform their patient populations about the best strategies to reduce cancer risk (1, 4, 6, 9, 19). In our pilot study, we sought to implement culinary medicine education, targeted to cancer risk reduction, into the education of medical students at the Case Western Reserve University (CWRU) School of Medicine, in hope that this training would inform the practices of future physicians and encourage them to pass this nutritional knowledge onto their patient populations, thereby reducing cancer risk in the US.

## 2 Materials and methods

### 2.1 Study design

**Figure 1:**
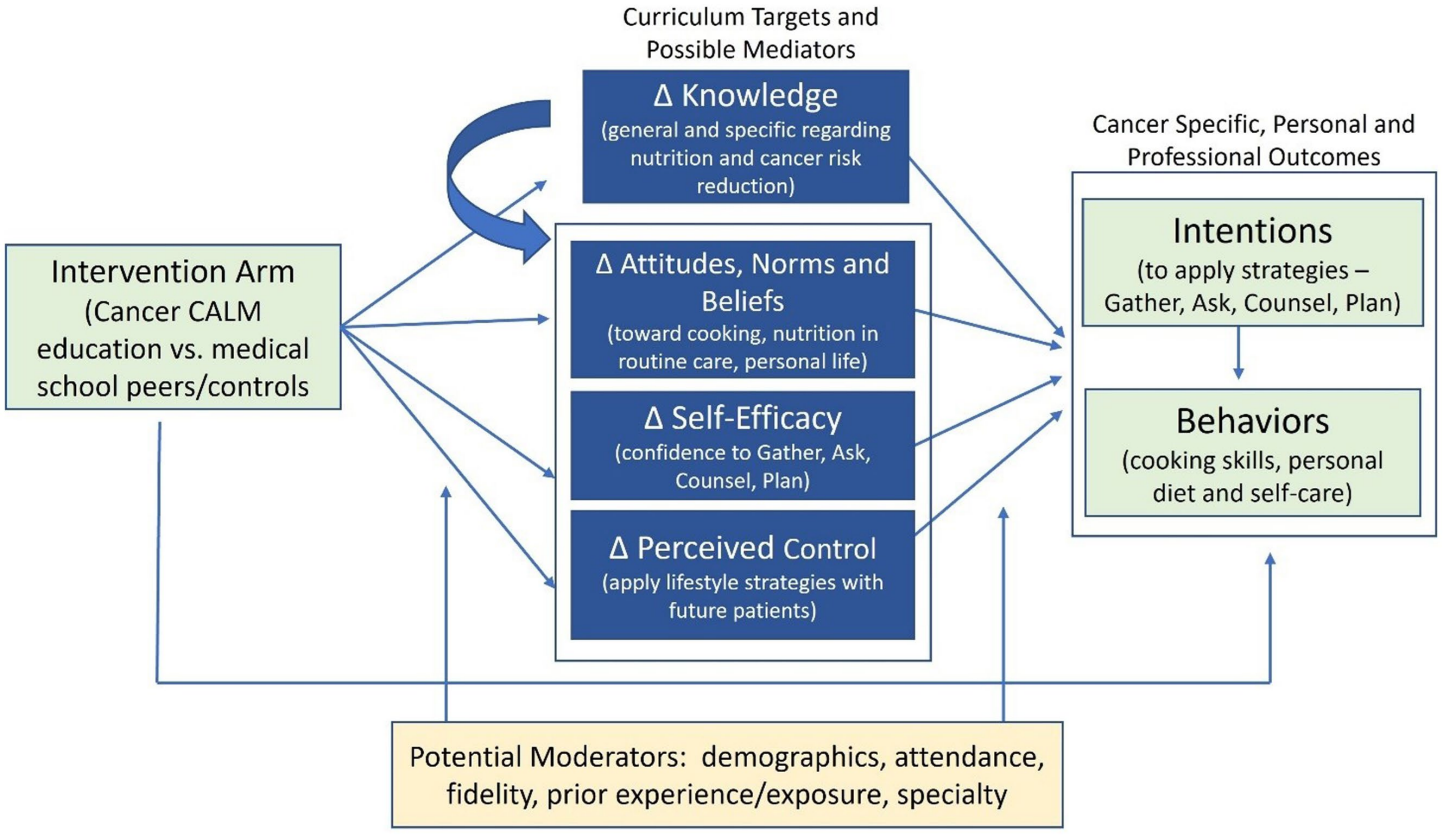
Framework for evaluating cancer CALM curriculum.

Guided by the Integrated Behavioral Model (34) and the Social Cognitive Theory (35, 36) and using a non-randomized two group design with pre-and post-test assessments, this pilot study examined the impact of the Cancer CALM (culinary and lifestyle medicine) curriculum on medical students’ cancer and nutrition knowledge, attitudes and beliefs, self-efficacy, and perceived control around dietary modification to prevent cancer, and their intentions of integrating what they have learned about diet and cancer risk reduction (CRR) into both their personal lifestyle and their future interactions with patients. This pilot study was approved by the Institutional Review Board at Case Western Reserve University in September of 2023. As shown in Figure 1, the underlying framework suggests that the intervention will directly affect the intention and behavior outcomes and potentially be mediated by the intervention targets of knowledge, attitudes, self-efficacy, and perceived control. Changes in the intervention targets will be associated with changes in intentions and behavioral outcomes.

### 2.2 Study participants

The pilot study population consisted of 86 first-year (M2) CWRU medical students, including 30 of the 31 students enrolled in the Jack, Joseph, and Morton Mandel Wellness and Preventive Care Pathway who participated in the seven 3-h education sessions focused on cancer risk reduction (CALM students) and an oversampling of 55 non-CALM M2 medical students (Controls) recruited via email (24, 37, 38). Oversampling was a precaution for a lower anticipated retention rate among controls, particularly among students beyond their first year of the pilot study. At the end of the curriculum, all 30 CALM students and 48 (87%) of the controls completed the post-test.

### 2.3 Intervention description

Each of seven, 3-h educational sessions included a ~ 45-min lecture on a key target area for CRR through diet and lifestyle changes, followed by a ~ 30-min learning activity to translate the education provided into potential patient care plans or patient conversations. The topics covered included prevention in practice and at home, dietary patterns, gut health, inflammation, metabolic imbalances, hormonal dysfunction, and environmental exposures. Then, ~90 min was spent cooking and utilizing culinary techniques, such as substituting high-risk ingredients and incorporating foods containing bioactive ingredients shown to mitigate cancer risk, into everyday recipes to practice implementing the nutrition education and care plans that could be recommended to future patients.

### 2.4 Data collection

All data were collected using Redcap. CALM students and controls were recruited via a confidential email from Redcap with details of the pilot study and links to the informed consent. Upon consent, participants completed the baseline survey approximately 2–3 weeks before the beginning of the Cancer CALM curriculum in September 2023 and the post-test within 2 weeks of the final Cancer CALM session in March 2024. To incentivize survey completion, study participants received a $25 Amazon gift card each time they completed a survey. Through Redcap, participants remained anonymous while still allowing matched repeated data collection. Anonymity was maintained through an auto-generated record #ID for each participant, ensuring their privacy while still enabling the tracking of responses over time.

### 2.5 Measures

As outlined in the conceptual framework (Figure 1) and described in detail below, we examined the impact of the curriculum on cancer and nutrition knowledge, attitudes and beliefs, and perceived control around dietary/lifestyle modifications to reduce cancer risk and intentions of integrating learned concepts about diet and CRR into both personal lifestyle and in future interactions with patients.

Cancer and nutrition knowledge was assessed with a derived index comprised of 15 multiple choice questions focused on overall cancer knowledge and current incidence statistics, as well as the biological mechanisms influenced by diet and lifestyle that are linked to cancer risk, and the key recommendations for CRR among them. Each correct answer received a point, resulting in an index range of 0–12.

Attitudes and Beliefs: *Attitudes toward Cooking* were assessed with the 4-item Negative Cooking Attitude subscale of the Cooking with a Chef (CWC) tool using a 5-point Likert scale (alpha = 0.857), from “strongly disagree” to “strongly agree” with items such as “I do not like to cook because it takes too much time” (39). Items were reversed for higher scores to represent positive cooking attitudes. The *Nutrition in Routine Care* 8-item subscale of the Nutrition in Patient Care Survey (NIPS) (40) was used to assess students attitudes toward including nutrition in routine patient care, rating their agreement on a similar 5-point scale on items such as “nutrition counseling should be part of routine care by all physicians, regardless of specialty.” Higher scores reflect more positive attitudes toward integration of nutrition into routine medical care.

Self-Efficacy: To assess students confidence in their ability to conduct cancer-specific assessments and counseling behaviors with their patients, we developed a five item scale (alpha = 0.840) that involved increasing levels of engagement with patients, asking them how confident (5 pt. scale, not at all confident to highly confident) they are that they can: (1) GATHER the necessary information needed to identify a patient’s risk of cancer, (2) ASK patients questions about their nutrition, dietary patterns, lifestyle and environmental exposures needed to assess cancer risk; (3) COUNSEL patients on ways they can modify their diet, lifestyle, and environmental exposures to reduce their cancer risk; (4) MAKE A PLAN with a patient to address their modifiable risks, including consultation with other health care providers (e.g., RDN) or referral to a lifestyle modification program; and (5) FOLLOW UP with a patient regularly to assess progress on the plan.

Perceived Control: To assess the students perceptions of how much control they will have in applying nutrition and risk reduction strategies with patients, we asked them to rate their level of agreement on the same 5-pt Likert scale (alpha = 0.513) as the attitude measures, on three questions: “I believe that I will have time to integrate what I’ve learned about nutrition and lifestyle changes into my patient care,” “I believe that the current health care system supports physicians in applying nutrition and lifestyle modification approaches with their patients” and “In practice, time with patients is very limited and beyond the control of most physicians (reverse coded).” The higher the score, the more perceived control.

Behavioral Intentions: To assess students intentions to apply cancer-specific risk reduction strategies with their patients, we used the same five assessment and counseling behaviors listed for self-efficacy above (i.e., Gather, Ask, Counsel, Make a Plan, Follow Up), however reframed the question stem to be “how certain are you that you will regularly engage in the following behaviors when you begin working with patients,” on a 7-pt scale from “Certain/practically certain” to “No chance or almost no chance” (alpha = 0.941).

Behaviors: As the second-year students do not yet have extensive exposure to one-on-one interactions with patients, we limited the behavioral outcomes to personal cooking skills and health behaviors. *Cooking Skills* were assessed using Lavalle et al. (41) 14-item Cooking Skills tool where students rated their skill level on a 7-point Likert scale of “very poor” to “very good,” on a wide range of cooking skills such as chopping, mixing, and stirring foods to baking goods such as cakes or bread, to preparing and cooking raw fish (39, 41). If students stated they had never done an activity/cooking skill, the response was coded at the midpoint of “neither good nor poor’.

Student’s *personal health behaviors* included a multi-item diet quality measure and single items that were dichotomized to document frequency of tobacco use (1 = not a user), alcohol use (1 = <3 drinks per week), and physical activity (1 = 3 + times per week). To assess diet quality, 12 items of the 14 Mediterranean Diet Assessment Tool (39) were used to assess the consistency of the individual’s dietary pattern with the Mediterranean dietary pattern. Due to the changing literature on the advantages/disadvantages of alcohol use and the rare use of sofrito in our student population, these items were not asked. The scoring rubric recommended by Martınez-Gonzalez et al. (39) was used to summarize diet quality, with a higher score reflecting higher diet quality. The resulting scale ranged from 0 to 12.

### 2.6 Statistical analyses

Baseline measures were examined using frequencies and descriptives, with group differences examined with t-tests and chi-square analyses. To determine the impact of the Cancer CALM curriculum on the curriculum targets (changes in knowledge, attitudes, efficacy, perceived control) and outcomes (changes in intentions, skills, behaviors), univariate general linear models were estimated, with group assignment as the fixed effect, the baseline measure of the outcome and any significant group differences at baseline are included as covariates. That is, for each curriculum target or outcome, the post-test measure (e.g., post-test knowledge) is entered as the dependent variable, with its baseline measure (e.g., pre-test knowledge) entered as a covariate, thus estimating the residual change in the outcome associated with the group assignment.

## 3 Results

### 3.1 Demographics and baseline measurement

Table 1 provides the baseline characteristics of the study population (n = 78). The average age was 24.3 years, and 37.2% were male. Most students reported their race as Asian (47.4%) or Caucasian (38.5%), followed by 7.7% Latino/Hispanic, 2.6% Black, and 3.9% other. While the two groups did not differ by age or race, there were significantly more males in the CALM group (53.3%), compared to the controls (27.1%) (*p* = 0.02). On average, CALM students attended 4.3 of the seven, three-hour education sessions. Seventy percent (21 out of 30) attended four or more sessions; 30% attended 6 or 7 sessions. Also of note, among the control students, those who completed the follow-up survey did not differ from those who did not on any of the baseline characteristics.

**Table 1:** Baseline characteristics of study population: total and by intervention group.

Baseline characteristics	Total (*n* = 78)	CALM students (*n* = 30)	Control students (*n* = 48)	*χ*^2^ or t, *p*-value
Demographics				
Age (M, SD)	24.3 (1.50)	24.3 (1.64)	24.4 (1.35)	−0.03; *p* = 0.98
Gender (% male)	37.2%	53.3%	27.1%	5.45; *p* = 0.02
Race/Ethnicity				
Asian	47.4%	63.3%	37.5%	8.53; *p* = 0.13
Hispanic/Latino	7.7%	3.3%	10.4%	
Caucasian	38.5%	26.7%	48.8%	
African-American/Black	2.6%	3.3%	2.1%	
Other	3.9%	3.3%	4.2%	
Prior nutrition related coursework (% Yes)	44.9%	56.7%	37.5%	2.74; *p* = 0.09
Cooking frequency (prepare more than half of meals at home)	62.8%	46.7%	72.9%	5.45; *p* = 0.02
Wellness behaviors No tobacco Exercises 3 + times/week Alcohol (less than 3 drinks/wk) Healthy Diet *(7 + on Med DietScore, range 1–12)*	98.7% 71.7% 89.7% 43.6%	96.7% 70.0% 90.0% 36.7%	100% 72.9% 89.6% 47.9%	No group differences
Cancer and nutrition knowledge at baseline (range 0–15)	6.51 (2.38)	7.10 (2.41)	6.15 (2.31)	−1.74; *p* = 0.09
Attitudes toward nutrition in medical practice (range 1–5)	3.91 (0.41)	3.98 (0.35)	3.86 (0.43)	−1.15; *p* = 0.25
Perceived control of implementing strategies in practice (range 1–5)	2.45 (0.42)	2.45 (0.47)	2.45 (0.40)	−0.02; p-0.99
Behavioral efficacy to implement cancer-specific strategies in practice (range 1–5)	3.21 (0.72)	3.03 (0.64)	3.31 (0.74)	1.74; *p* = 0.09
Intentions to implement cancer-specific strategies in practice (range 1–7)	4.95 (1.29)	5.10 (1.18)	4.87 (1.35)	−0.81; *p* = 0.42

Regarding other characteristics, the two groups did not differ significantly on any intervention targets (i.e., knowledge, attitudes, perceived control, self-efficacy) or behavioral intentions at baseline. Similarly, the groups did not differ on wellness behaviors, such as smoking, alcohol use, exercise, and diet. However, they did differ with regard to how often they prepared home-cooked meals, with the control group home-cooking three-quarters of their meals on average, compared to the intervention group home-cooking half of their meals on average (*p* = 0.02). Lastly, while not reaching statistical significance, CALM students were more likely to have prior nutrition coursework than the controls (56.7% vs. 37.5%) but had somewhat lower confidence (i.e., efficacy) to implement CRR strategies at baseline than their peers (3.03 vs. 3.31) (both *p* = 0.09).

### 3.2 Intervention impact

To determine the impact of the Cancer CALM educational experience on the curriculum targets and outcomes over time, we estimated the residual effect (i.e., change) of each outcome by controlling for the baseline status of that variable, as well as gender and cooking frequency at baseline due to group differences. Table 2 provides the estimated marginal means at post-test for the five curriculum targets (knowledge, attitudes, efficacy and perceived control) and three outcomes (behavioral intentions, cooking skills, dietary quality) and the between-subjects effects and statistics associated with group association in the estimated change.

**Table 2:** Estimated marginal means at post-test and between subjects effects, representing residual change in curriculum targets and outcomes (pre-post) for cancer CALM vs. controls.

Curriculum targets and outcomes	CALM students (*n* = 30)			Control Students (*n* = 48)			Between Subjects effect of group	
	Est. marginal mean at post-test*	SE	95% CI	Est. marginal mean at post-test*	SE	95% CI	*F* statistic	*P* value
Cancer and nutrition knowledge (0–15)	7.98	0.447	(7.1,8.9)	6.53	0.347	(5.8,7.2)	6.06	0.016
Attitudes toward nutrition in practice	4.12	0.067	(3.9,4.3)	3.93	0.052	(3.8,4.0)	4.43	0.039
Cooking attitudes	3.66	0.074	(3.5,3.8)	3.58	0.058	(3.5,3.7)	0.65	0.424
Perceived control	2.48	0.099	(2.3,2.7)	2.34	0.077	(2.3,2.6)	1.20	0.276
Behavioral efficacy	3.88	0.104	(3.7,4.1)	3.28	0.081	(3.2, 3.4)	19.54	<0.001
Behavioral intentions	4.99	0.209	(4.6,5.4)	4.77	0.163	(4.5,5.1)	0.64	0.427
Cooking skills	5.23	0.130	(5.0,5.5)	5.34	0.101	(5.1,5.5)	0.41	0.525
Diet quality (range 1–12)	6.37	0.395	(5.6,7.2)	5.67	0.286	(5.1,6,2)	1.94	0.168

*Based on univariate linear model with group as fixed effect, and baseline measure of indicator, gender, and cooking frequency at baseline at covariates.

Compared to the controls, students receiving the Cancer CALM curriculum reported greater improvements in cancer and nutrition knowledge (7.98 vs. 6.53; *F* = 6.06; *p* = 0.016), attitudes toward nutrition as part of the clinical practice (4.12 vs.3.93; *F* = 4.43; *p* = 0.039), and their self-confidence (i.e., behavioral efficacy) in integrating nutrition assessment, counseling, planning, and follow up in their clinical practices (3.88 vs. 3.28; *F* = 19.54; *p* < 0.001), compared to controls. Another notable difference was in diet quality where CALM students reported larger increases in their compliance to the elements of the Mediterranean diet, although not reaching statistical significance (6.37 vs. 5.67; *F* = 1.94; *p* = 0.17). There were no group differences observed in cooking attitudes, perceived control, behavioral intentions or cooking skills.

To better understand the substantial changes that were observed in the students’ behavioral efficacy over time, we took a closer look at the clinic-focused behaviors that make up the scale. The students were asked about their confidence in engaging in five different activities with their future patients from gathering information to asking patients about their diet and lifestyle practices, to counseling, making a plan, and following up on ways to modify patient behaviors and reduce cancer risk.

As shown in Table 3, the CALM students reported significant increases in confidence across all five of the items, while the controls reported little to no change over time. The largest impact was observed in the confidence around counseling patients on ways to reduce their risk and exposure to cancer risk factors and confidence around making an actual plan with their patients, including making referrals to other health care providers or lifestyle modification programs.

**Table 3:** Changes observed in behavioral efficacy items for cancer CALM students vs. controls.

Behavioral efficacy items	CALM students (*n* = 30)			Control students (*n* = 48)		
	Pretest	Posttest	Difference	Pretest	Posttest	Difference
**GATHER** the necessary information needed to identify a patient’s risk for cancer?	3.03 (0.72)	3.47 (0.68)	0.44*	3.17 (0.88)	3.33 (0.81)	0.16
**ASK** patients questions about their nutrition, dietary patterns, lifestyle, and environmental exposures needed to assess their cancer risk	3.40 (0.86)	3.97 (0.72)	0.57**	3.58 (0.90)	3.58 (0.82)	0.00
**COUNSEL** patients on ways they can modify their diet, lifestyle, and environmental exposures to reduce their cancer risk.	2.73 (0.98)	3.73 (0.74)	1.00***	3.00 (0.88)	3.04 (0.87)	0.04
**MAKE A PLAN** with a patient to address their modifiable risks, including consultation with other health care providers (e.g., dietitian) or referral to a lifestyle modification program (e.g., smoking cessation, exercise class).	2.73 (83)	3.77 (0.68)	1.04***	3.13 (0.98)	3.19 (0.94)	0.06
**FOLLOW UP** with a patient regularly to assess progress on the plan.	3.27 (83)	3.97 (0.67)	0.70***	3.48 (1.01)	3.54 (90)	0.06

*p < 0.05; ** < 0.01 ** < 0.001.

## 4 Discussion

After completion of the curriculum, CALM students showed significant improvement over the controls in cancer and nutrition knowledge scores, attitudes toward nutrition in clinical practice, and confidence/self-efficacy in integrating CRR strategies in patient care (assessment, counseling, planning, follow-up). Differences in attitudes toward cooking, perceived control, behavioral intentions, and diet quality were not significantly different between groups; however, it is worth noting that dietary adherence to the Mediterranean dietary pattern (as evidenced by the diet quality measurements) was improved in the CALM students compared to the controls (though it did not reach statistical significance). Future physician adherence to the Mediterranean dietary pattern is worth noting because research has demonstrated that patients are more likely to receive quality and frequent nutrition advice from physicians who also implement healthful dietary patterns in their own lives (27, 42, 43).

The data evaluating confidence/self-efficacy in integrating CRR strategies in patient care, changes in the students’ perceived control, and self-efficacy toward culinary medicine and preventive healthcare showed no significant difference in behavioral efficacy between pre-and post-test surveying of the controls. However, there were significant differences in the perceived control and efficacy of CALM students toward implementing this nutrition knowledge in the care of their patients. The most significant difference was observed in the students’ enhanced perception of their ability to counsel patients on how to incorporate cancer risk reduction strategies into their lifestyle and their ability to plan with patients to address their specific modifiable cancer risks. These findings are consistent with previous studies and reviews demonstrating that CM interventions can improve future providers’ confidence and skills in providing nutritional support (26, 27, 29), particularly compared to more traditional, didactic approaches to teaching nutrition (44, 45). Moreover, this knowledge/attitude change predicts that the future providers will likely implement this knowledge into their practices (27).

Nevertheless, despite these positive findings, the paradox of increased confidence/self-efficacy yet minimal changes in behavioral intentions to implement CRR strategies into practice is noteworthy and presents a unique challenge to medical schools that seek to implement CM interventions into their curriculum (26, 29). One factor contributing to lack of improved intentions to implement CRR strategies into clinical practice may be the expected “time pressures” experienced by physicians in modern patient care (30, 46, 47). The average follow-up visit to a primary care appointment with patients lasts roughly 18–20 min and requires physicians to discuss an array of topics (47, 48). This leaves physicians feeling the pressure of this limited time, especially when dealing with complex cases or new patients (46). Educating providers to include CRR strategies only complicates these already-packed appointment slots. On average, health care providers would need 14.1 h per day to discuss and provide all of the various preventive care measures recommended by the United States Preventive Services Task Force (USPSTF) and the Advisory Committee on Immunization Practices (ACIP), and this time does not include counseling on chronic disease and acute care, along with documentation before and after appointments (48). Moreover, it is worth noting that from the point of pre-testing to post-testing, the CALM students would have received almost another year of clinical practice exposure, which may have changed their views potentially contributing to a negative shift in their intentions to implement CRR strategies.

While the Cancer CALM education program cannot relieve the time pressures experienced by physicians (46–48), our programming aims to address this barrier by including activities designed to give future physicians new ways to communicate CRR strategies with future patients quickly and efficiently. Our program features scripted, hypothetical patient care conversations targeted toward various specialties to demonstrate that conversations about CRR can be integrated seamlessly and efficiently in practices ranging from primary care to orthopedic surgery, despite the specificity of subspecialties (27, 42, 43). These efforts could also help to close the “counseling gap” among physicians in surgical specialties that fall short in preventive care counseling efforts compared to non-surgical physicians (42).

In the US, nutrition education at the MD/DO level is subpar, with only 29% of medical schools providing students with the recommended 25 h of nutrition-focused education in 2023 (26). However, there is a growing interest in strengthening nutrition programming, as evidenced by an increase in nutrition electives, service-learning opportunities, specialty tracks, and interest groups provided by medical schools, in addition to community cooking and CM programming (21, 27, 28). This interest in enhancing nutrition education for providers and patients alike is recognized at the federal level, with the Biden-Harris Administration hosting the first White House Conference on Hunger, Nutrition, and Health in 50-plus years and highlighting the need for “food as medicine” interventions (49, 50).

While the CWRU School of Medicine Cancer CALM program may be the first CM education intervention to target cancer risk reduction at the medical student level (21, 26, 44, 45), numerous programs seeking to educate the public and providers alike on lifestyle and dietary choices for cancer risk reduction have taken root. Culinary medicine is uniquely situated to promote CRR strategies due to the community and cooking-skills-based approach that can help promote psychosocial well-being, enhance confidence in the kitchen, and improve nutrition counseling for patients, demonstrating that these offerings can benefit clinicians and patients alike (26, 28, 29, 33, 51). Following the guidelines put forth by the American Cancer Society (6, 7), programs such as “Cooking After Cancer” have been evaluated to determine their effectiveness (28). The American Institute for Cancer Research (AICR) has implemented a survivor-targeted program, “Coping with Cancer in the Kitchen,” that utilizes the skills of RDNs and social workers to successfully empower patients to engage in best health and food practices during 90-min educational sessions (33, 52). While this program and “Cooking After Cancer” both target survivorship, the CM-based strategies provide much of the same fundamental cancer risk reduction information as the CWRU Cancer CALM program, but just at the survivorship level (28, 33). The American Cancer Society’s “Coping with Cancer in the Kitchen” showed a significant increase in participants’ understanding of the importance of a plant-based diet and improved confidence to prepare and follow this dietary pattern (33). Similarly, the Sustainable Food Center in Austin, Texas offers a similar cooking-class-style program for survivors called “Cooking After Cancer;” however, the same significant changes in confidence and behavior were not observed, as many participants were previously engaging in healthy eating practices (28, 33). Nevertheless, for patients to reap these benefits, providers must first have the knowledge base and confidence to educate their patient populations on this material.

Culinary medicine provides a hands-on means to educate providers on strategies to help patients and help themselves decrease their risk for chronic illness through dietary and lifestyle interventions, making it one of the best options for nutrition education targeting the lifestyles of both future physicians and their future patients (20, 21, 27, 53). Between 2012 and 2020, 34 CM programs were implemented at medical schools across the country (52); however, to date, none of these programs specifically target CRR– making the CWRU Cancer CALM program the first of its kind (21, 26, 44, 45). Additionally, most of these CM programs are elective in nature, and the curricula are unstandardized (44, 45), with most medical schools developing their own curriculum while others follow Tulane’s Health Meets Food program (52).

As a pilot, there are limitations to the study. First, this pilot study had a relatively small sample size from one medical school (30 CALM students, 48 controls), thereby limiting the power of the study to detect small group differences and influencing the ability to generalize these findings (29). Moreover, we did document baseline differences between the intervention and control groups, namely a difference in gender composition and current home meal preparation, which may be explained by male students and those with less cooking experience choosing the Wellness Pathway as a way of improving their own personal wellness. However, the analyses did control for these differences, and it should be noted that propensity score matching was applied to the data, but did not change the results and thus, the decision was to retain the larger sample size. A third limitation was that attendance was sporadic due to the demanding schedules of the medical students; therefore, it is possible that more significant positive changes could have been observed in the pre-and post-testing period if the students evaluated received the full curriculum (28, 33). In the future, attendance may be improved by making it mandatory.

Regarding personal health of the medical students, there was little change documented in the wellness measures, outside of the slight, but not significant, improvement in diet quality of the CALM students. However, without objective measures such as changes in central adiposity or inflammatory markers, determining the impact of this intervention on the personal health of the medical students was a limitation in this pilot study (11–13, 15, 17, 33). Additionally, compared to the CALM students, the control group reported significantly more at-home cooking frequency at baseline. So observing a significant improvement in at-home cooking and other wellness behaviors following the intervention was inherently unlikely (32). Another limitation of this pilot study is the lack of follow-up in a clinical setting (29). Medical education is arduous and time-consuming, with most medical students completing roughly seven years of training before serving as an attending physician. Therefore, it will be several years before the 2nd year medical students even begin implementing the knowledge gained in the Cancer CALM education intervention in their own practices, making it challenging to objectively determine if the students have applied the skills gained in a clinical setting (32). Moreover, while the survey tools utilized in this study are validated tools (39–41, 54–58), the limitations previously mentioned related to attendance (28), time burdens (46–48), and lack of direct clinical implementation (29), may affect the tools’ ability to assess the intervention adequately. Research surrounding CM education interventions at medical institutions remains relatively new; therefore, various methods (qualitative and quantitative) could be utilized to collect and interpret results. However, qualitative strategies remain a focus (52).

CM education for future physicians opens a whole new world of opportunities to enhance the quality of patient care along with the health and well-being of physicians (26, 27, 29, 33). Nevertheless, for this impact to be felt on a broader scale, more medical schools need to implement CM education as integral parts of their curriculum. To do this, standardizing the education provided is an essential first step (44, 45, 52). Additionally, given the time pressure burdens felt by physicians (46–48), increased public health action is needed to regulate food corporations and incentivize healthy dietary choices so that nutrition education is not solely reliant on overworked healthcare providers (50).

## Data Availability

The de-identified raw data supporting the conclusions of this article will be made available by the authors, upon request.
